# Effects of Extra Virgin Olive Oil and Apples Enriched-Dark Chocolate on Endothelial Progenitor Cells in Patients with Cardiovascular Risk Factors: A Randomized Cross-Over Trial

**DOI:** 10.3390/antiox8040088

**Published:** 2019-04-04

**Authors:** Francesca Felice, Alessandra Francini, Valentina Domenici, Mario Cifelli, Ester Belardinelli, Luca Sebastiani, Claudio Cantini, Rossella Di Stefano

**Affiliations:** 1 Cardiovascular Research Laboratory, Department of Surgical, Medical and Molecular Pathology and Critical Care Medicine, University of Pisa, 56100 Pisa, Italy; ester.belardinelli@gmail.com; 2 BioLabs, Institute of Life Sciences, Scuola Superiore Sant’Anna, 56127 Pisa, Italy; a.francini@santannapisa.it (A.F.); luca.sebastiani@santannapisa.it (L.S.); 3 Department of Chemistry and Industrial Chemistry, University of Pisa, 56100 Pisa, Italy; valentina.domenici@unipi.it (V.D.); mario.cifelli@unipi.it (M.C.); 4 Trees and Timber Institute, IVALSA-CNR, Sesto Fiorentino, 50019 Florence, Italy; cantini@ivalsa.cnr.it; 5 Interdepartmental Research Center “Nutraceuticals and Food for Health”, University of Pisa, 56100 Pisa, Italy

**Keywords:** dark chocolate, endothelial progenitor cells, cardiovascular risk factors, metabolism

## Abstract

Background: Endothelial dysfunction has been associated to cardiovascular outcomes in patients with cardiovascular risk factors. Circulating endothelial progenitor cells (EPCs) play an important physiological role for their reparative potential of vascular integrity, but are numerically reduced and functionally impaired in patients with cardiovascular risks. This study assesses the effects of Extra Virgin Olive Oil (EVOO) and apple-enriched dark chocolate intake on the blood levels of EPCs. Methods: Thirty volunteers with cardiovascular risk factors, enrolled in a randomised, crossover, four-weeks trial, received a solid dark chocolate bar (40 g/day) containing 10% EVOO or 2.5% dry apples. Urine samples were analyzed for endogenous metabolites. Circulating EPCs levels, clinical data and anthropometric examinations were collected. Results: 26 volunteers (M/F:14/12, 51 ± 9 years of age) completed the study. Comparison of pre-post intervention revealed a significant increase in EPCs levels associated with EVOO-dark chocolate consumption. Most biochemical parameters were not significantly modified by both chocolates. Conclusions: This study shows that a daily consumption of a non fattening dose of dark chocolate enriched with EVOO improves blood levels of EPCs, a well known surrogate biologic marker for endothelial function.

## 1. Introduction

Endothelial dysfunction has been associated with cardiovascular disease (CVD). Lifestyle modifications such as nutrition and exercise represent protective measures to reduce the risk of CVD [1]. Bone marrow-derived endothelial progenitor cells (EPCs) [2] are a new marker of endothelial dysfunction [3]. In particular, circulating EPC levels have been identified as a surrogate biologic marker for vascular function in the general population [4,5]. These cells have the ability to influence adult vasculogenesis in areas with reduced oxygen supply [6] or to stimulate the re-endothelialization of injured blood vessels [7].

The relationship between EPCs and cardiovascular risk factors has also been extensively investigated [8,9]. Risk factors for CVD are associated with a blunted capacity for repair of the endothelial damage and a reduction of circulating EPCs and several studies have given the role of a biomarker of prognostic value to the number of EPCs, resulting in an independent predictor of clinical outcomes [4,10]. Moreover, it has been demonstrated that healthy lifestyles increase the number of circulating EPCs [11], including diets rich in polyphenols [12].

Cocoa products are among the richest sources of polyphenols in our diets and consumption of cocoa products has been related to health benefits including for the cardiovascular system [13,14,15,16]. Many studies have demonstrated the potential health implications of dark chocolate constituents. Due to the high levels of flavonoids (epicatechin, catechin and their oligomers), the consumption of cocoa has been associated to the improvement of endothelial function measured as brachial artery flow-mediated dilation (FMD) in several clinical studies (mostly revised by Ludovici et al.) [17,18,19]. Since there is a strict direct correlation between the EPC number and FMD [4], it is of great interest to study EPCs modulation induced by different dark chocolates.

Several studies have also noted beneficial effects on human health from extra virgin olive oil (EVOO) as well as apples [20,21,22].

Dietary preferences, life style and genetics may influence an individual metabolic phenotype, health status and susceptibility to develop diseases [23]. Detailed profiling of metabolic status can provide insights into the mechanisms underlying cardiovascular diseases. Therefore, the evaluation of circulating metabolites associated with the cardiovascular system has great potential.

Aims of the present study were to develop a new set of cocoa (70%) bars containing top-quality EVOO and dried apples produced by old autochthonous cultivars, and to evaluate the endothelial function, as medians increase of circulating EPCs, after cocoa bar intake with a daily intake of 40 g of enriched-dark chocolate, over a period of 4 weeks, in healthy individuals carrying at least 3 cardiovascular risk factors, enrolled from the Tuscany region. EPC were identified as circulating CD133+CD34+ KDR+ cells. Proton nuclear magnetic resonance (^1^H NMR) spectroscopy was used for monitoring metabolic changes in the urine. Our hypothesis is that the consumption of cocoa would improve endothelial function (expressed as increase in EPCs number) and that the researching of an endogenous metabolic biomarker could identify new pathways involved in cocoa benefits.

## 2. Materials and Methods

### 2.1. Participants

Male and female Caucasian Italian volunteers, from the Tuscan region, with at least three cardiovascular risk factors from smoking, dyslipidemia, hypertension, overweight and family history for cardiovascular disease, aged 25 to 65 years, were recruited. Exclusion criteria included eating disorders, no history of cardiovascular disease, diabetes mellitus, sleep apnoea, inflammatory gastrointestinal tract disease, pregnancy, use of anti-inflammatory medication, restricted diets by choice (i.e., vegan, carbohydrate-restricted, etc.), or a known allergy to cocoa or chocolate. 

The study protocol (number 151-2015) was approved by the local Ethics Committee (Full name: “Comitato Etico per la Sperimentazione Clinica Area Vasta Nord Ovest c/o Azienda Ospedaliero-Universitaria Pisana (AOUP), Pisa”). All volunteers were informed about the study and all subjects provided their written informed consent to participate in the study. Data were managed blindly.

### 2.2. Recruitment, Screening and Study Design

**Figure 1:**
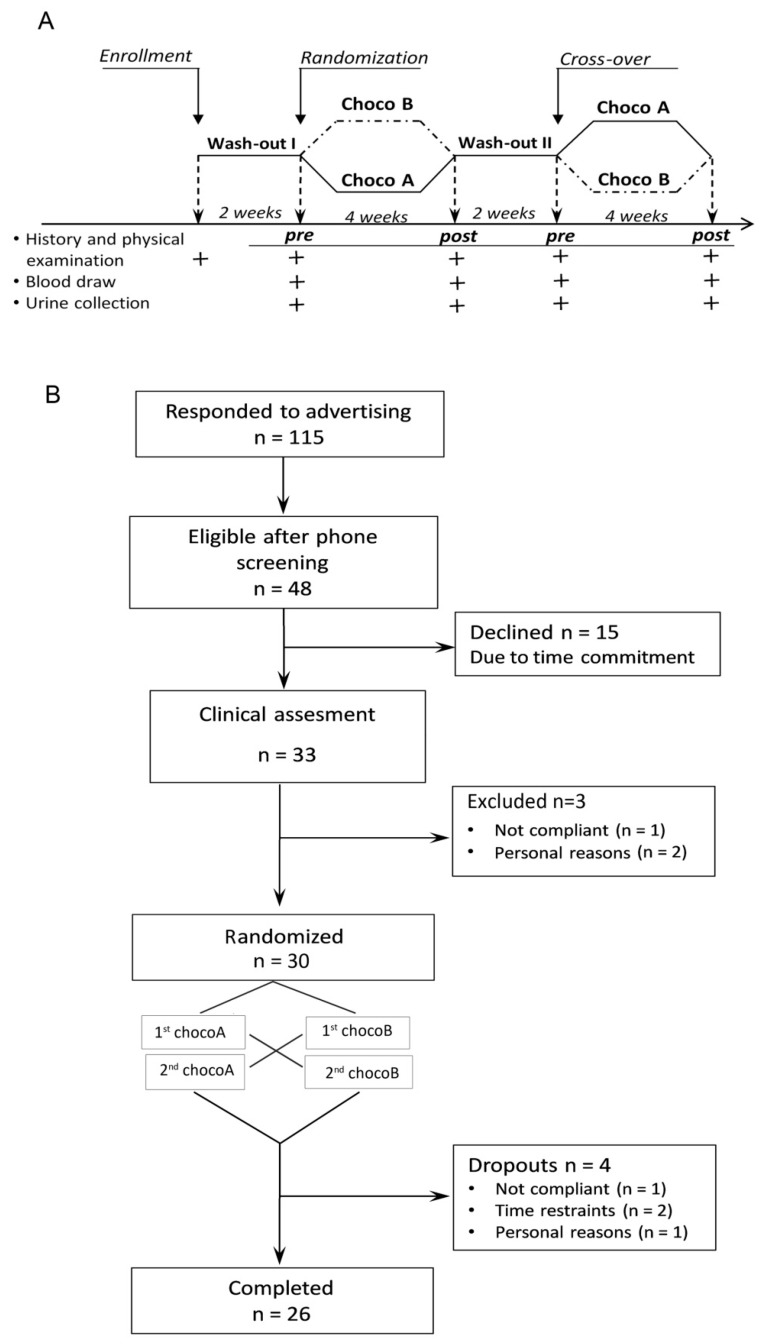
(**A**) Study protocol and (**B**) study flow diagram.

Participants were recruited from March 2015 to July 2016 at the University of Pisa. One hundred and fifteen potential participants expressed interest in the study. A telephone screening was conducted to evaluate the inclusion criteria. Forty height volunteers were scheduled for a screening appointment at the Cardio Angiology Unit of the AOUP of Pisa. Thirty-three subjects were recruited for clinical assessment (Figure 1).

The study was a randomized, single-blind, cross-over trial that involved the consumption of a solid dark chocolate bar (40 g/day) containing 10% EVOO and a matched solid dark chocolate bar containing 2.5% dry Panaia Red apples over 28-day period (4 weeks) with 2 weeks of wash-out between interventions (Figure 1) [19,24]. The circulating number of EPCs were the primary endpoints. Biochemical parameters and metabolites were the secondary endpoints.

The unit of randomization was the individual. Numerical designations treatments were assigned by an independent investigator. Chocolate treatments did not differ in size, color, or shape, and only minor differences in taste were noted. 

Volunteers enrolled in the study were asked to keep a regular life style with regular medication and dietary habits, and avoid only chocolate and derived-chocolate foods, vitamin supplements, coffee and non-steroidal anti-inflammatory drugs 2 weeks before starting the trial and throughout the entire study. Blood pressure, body mass index (BMI) and venous blood sample were taken after overnight fasting at each pre- and post-intervention. Samples were analyzed for EPCs, fasting plasma levels of circulating glucose and lipid profile (triglycerides, total cholesterol, high-density lipoprotein (HDL) cholesterol, and low density lipoprotein (LDL) cholesterol). Plasma was immediately processed and stored at −80 °C until analysis.

The nutrient composition of the chocolate bars is shown in Table 1.

### 2.3. Blood Pressure

Blood pressure was determined using the Omron automated blood pressure device (Omron Health care Europe B.V., Hoofddorp, Netherlands) in accordance with the guideline for blood pressure (BP) measurement [25].

### 2.4. Evaluation of Circulating Endothelial Progenitor Cells

The circulating EPCs were defined by the simultaneous expression of surface markers CD34/KDR/CD133 by flow cytometry analysis as already described, without any modification [26]. 

Validation of the assay was performed adopting the gating strategy defined by the International Society of Haematotherapy and Graft Engineering (ISHAGE) guidelines [27]. FACS-Calibur instrument was used for fluorescence analysis. Data were processed using Cell Quest Software (BD Pharmigen, Oxford, UK). Each analysis included at least 1,000,000 events. The circulating EPC levels were expressed as absolute number of cells per ml of blood.

### 2.5. Analytical Parameters of Ingredients and Final Bars

Chemicals, reagents and characterization of the extracts are reported in Supporting Information materials (Tables S1 and S2, Figure S1).

Antioxidant compounds were extracted from 7.5 g of dark chocolate containing 2.5% of dry Panaia apples and 10% EVOO respectively (*n* = 3) using 25 mL of 80% aqueous methanol.

Samples used for NMR analyses were added with D_2_O as a deuterium source for the field locking operation during the NMR experiments. The samples were vortexed at 1000 rpm for 30 min, and the supernatant extract was filtered with a 0.45 mL cellulose filter (Millipore, Milan, Italy) and kept at 20 °C.

Total phenolic content (TPC) of the 80% aqueous methanol extracts was analyzed adding in 30 µl of extract (or standard solution of gallic acid), 150 µl of Folin–Ciocalteu’s phenol reagent. The mixture was shaken and after 8 min, 600 µL of 10% (*w*/*v*) Na_2_CO_3_ solution and 3 mL of ultra-pure water were added and incubated at 20 °C for 120 min. Absorbance was measured at 765 nm and total phenolic contents were expressed as mg gallic acid equivalents (GAE) per 100 g of apple material [28].

The scavenging activity of 1,1-Diphenyl-2-picrylhydrazyl (DPPH) radicals was evaluated in both chocolates in 200 μL samples methanol extract and mixed with 800 μL of Tris-HCl 100 mM solution pH 7.0. One ml of methanolic solution of DPPH 250 µM was added to this mixture and the tubes were maintained for 30 min protected from light; absorbance was measured at 517 nm. Methanol was used as control. The percentage of DPPH inhibition was calculated using the following formula: Inhibition ratio (%) = [(absorbance of control − absorbance of sample) (absorbance of control)]. The IC_20_ values were calculated from the graph plotted as inhibition percentage against the concentration. IC 20%, meaning 20% of the DPPH radical, was the sample amount required to be scanned.

### 2.6. ^1^H NMR Analysis of Chocolate Extracts Samples

#### Sample Preparation and ^1^H Spectra Acquisition

A 400 mL aliquot of chocolate extracts was inserted in a 5 mm NMR tube and ^1^H NMR spectra were recorded on a Bruker Avance DRX 400 MHz spectrometer equipped with an ATM BBFO probe (Bruker Italia srl, Milano, Italy). Larmor frequency for the proton was 401.31 MHz, a field locking operation was performed on the heavy water signal and the proton 90° pulse was calibrated to 15 s. For each sample, 128 scans (64 K data points) were acquired, while a spectral width of 8012 Hz, an acquisition time of 2.00 s, a recycle delay of 3.00 s, and pulse length *t* = 5 s was chosen. Double Pre-saturation irradiation was used during the D1 recycling time in order to suppress solvent signals, namely water and methanol (power for irradiation 0.25 mW). NMR free induction decay (FID) signals were processed by applying a line-broadening factor of 0.3 Hz by using the Bruker Topspin software (version 3.1) (Bruker Italia srl, Milano, Italy). Thereafter, baseline and phase were corrected manually. For each chocolate, three samples have been analyzed for statistical analysis and signals of theobromine, catechin and epicatechin have been used to quantify these substances in the samples. Concentrations, expressed as mg/g have been evaluated from the integration of specific proton signals following the calibration curve procedure described in Francini et al. (2017) [28]; calibration curves and further details of the procedure are reported in the Supporting Information (Table S3 and Figure S2).

### 2.7. ^1^H NMR Analysis of Urine Samples

#### Sample Preparation and ^1^H Spectra Acquisition

The urine samples were thawed before analysis and centrifuged for 5 min at 12,000× *g*. A 500 μL aliquot of the supernatant was adjusted to pH 6.8 using 100 μL of a deuterated phosphate buffer solution (KH_2_PO_4_, final concentration of 0.2 M) into a 5 mm NMR tube.

The one-dimensional proton NMR spectra of urine samples, showing metabolic profiles of the subjects under study, were recorded with the same experiment and parameters previously described. In the present case, as only the water signal needed suppression, a single occurrence of pre-saturation irradiation was used during the D1 recycling time in order to suppress the water signal (power for irradiation 0.25 mW). The spectra were then processed similarly to that reported in Section 2.6 for chocolate samples analysis. Here, the ppm scale was referenced for a creatine signal at 3.05 ppm. 

The metabolic profiles acquired have then been investigated in terms of variations of the signal corresponding to specific endogen metabolites, related to cardiovascular risk [29,30,31], namely, carnitine, 2-hydroxyhyppurate, L-tyrosine and phenylalanine. The metabolites have been identified in the spectrum from characteristic signals as described in the Results section. 

### 2.8. Statistical Analysis

Means ± standard deviation was used to summarize continuous variables. Categorical data are presented by frequencies and percentages. One-sample Kolmogorov–Smirnov test was used to evaluate distributions of EPC levels. EPC values were log-transformed before being used as continuous variables in statistical analyses. Data obtained at baseline (pre-treatment) were compared with data obtained at the end of the study (post-treatment) by paired Student’s *t*-test or Wilcoxon test for variables with normal distribution and without normal distribution, respectively.

EPC number was defined as primary outcomes. Metabolite and biochemical parameter were the secondary outcomes. The sample size was based on information from the previous studies [24,32] and a number of 40 subjects was necessary. GraphPad PRISM 5.0 (GraphPad Software Inc., San Diego, CA, USA) was used for statistical analysis. *p* < 0.05 was considered statistically significant. Statistical analyses *t*-test for total phenolic content, DPPH and phenolic compounds determined by ^1^H qNMR in chocolate were done by using NCSS 2004 Statistical Analysis System Software (NCSS LLC, Kaysville, UT, USA). 

## 3. Results

### 3.1. Cocoa Bar Product Characteristics

Differences between the products have been observed. The two types of chocolates tested showed statistically significant differences for total polyphenols, demonstrating that not all types of chocolate are equal sources of these compounds. In particular, when specific polyphenols analyzed were investigated by NMR analyses, dark chocolate with 10% EVOO showed an increment of theobromine, catechin, and epicatechin up to 21, 25 and 27% compared to dark chocolate with 2.5% apple, respectively.

The antioxidant activity of chocolates demonstrated in DPPH IC_20_, showed that dark chocolate with 10% EVOO has the highest antioxidant activity, 3.8 times higher then chocolate with 2.5% apple (Table 2).

### 3.2. Clinical Outcome

Out of the 30 randomly assigned subjects, 26 completed the study (14 male and 12 women); there were 4 dropouts: 1 participant completed the baseline visit only and 3 participants dropped out after the first diet period. None of the patients experienced major adverse events, cardiovascular-specific events, or hospitalization during the study period.

Subjects had a mean age (±SD) of 51 ± 9 years and a body mass index or BMI (in kg/m^2^) of 29 ± 6. All individual medications and treatment paradigms remained unaltered throughout the study. Baseline characteristics are shown in Table 3. 

### 3.3. Endothelial Progenitor Cell Levels

**Figure 2:**
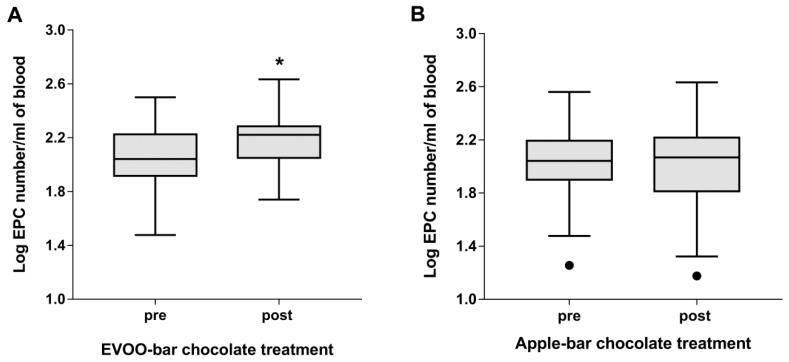
(**A**) Number of circulating endothelial progenitor cell (EPC) levels before and after 28 day of EVOO-dark chocolate bar consumption (40 g/day). Data are reported as Log transformed value of CD34+/KDR+/CD133+ EPCs number. * *p* < 0.05 (Tukey whiskers are reported). (**B**) Number of circulating EPC levels before and after 28 days of apple-dark chocolate bar consumption (40 g/day). Data are reported as Log transformed value of EPC number (Tukey whiskers are reported, black dots are values less than the lowest value represented by lower whisker).

At baseline, the EPC levels did not differ between the two groups. After 28-day EVOO-dark chocolate bar consumption, the circulating CD34+/KDR+/CD133+ EPCs number increased significantly compared with baseline (pre-treatment) (*p* < 0.05) (Figure 2A). 

The effect of Panaia red apple-dark chocolate bar consumption on circulating EPC levels is shown in Figure 2B. No significant change was observed after 28-days of treatment in the circulating CD34+/KDR+/CD133+ EPCs number. 

### 3.4. Biochemical Parameters

No statistically major clinical effects were observed in all participants after 28 days of both EVOO- and Panaia red apple-dark chocolate intake (Table 4 and Table 5). However, it is of interest that BMI and glucose remained unchanged in all participants.

After Panaia red apple-dark chocolate bar consumption, as shown in Table 5, although we observed a decrease in triglycerides levels (delta value, mean ± SD: −17.25 ± 62), LDL serum cholesterol increased after treatment (*p* = 0.03). However, post-hoc Bonferroni’s correction showed a *p* value of 0.29, demonstrating a random effect.

### 3.5. H NMR Spectrum of Urine

**Figure 3:**
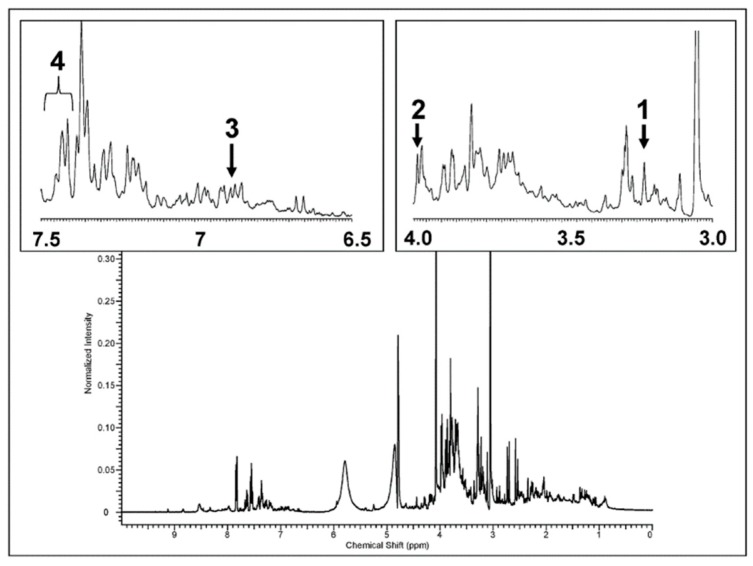
^1^H NMR spectrum of urine sample of one of the patient after the EVOO-enriched dark chocolate consumption, acquired as described in the text. Characteristic signal of the metabolites under investigation are reported in the insets as follows: 1. Carnitine, singlet at 3.23 ppm, 2. 2-hydroxyhyppurate, singlet at 3.99 ppm, 3. L-tyrosine, doublet at 6.88, 6.90 ppm, 4. Phenylalanine, multiplet at 7.41–7.44 ppm.

In order to evaluate a qualitative level of the main metabolites strictly correlated with the cardiovascular system, usually defined as “biomarkers of exposure”, the urine samples of patients before and after the treatment were characterized by ^1^H NMR spectroscopy. We decided to focus on the EVOO-enriched dark chocolate consumption due to its positive effect on EPCs. A typical ^1^H NMR spectrum of urine sample, acquired as described in the “Methods” section, is reported in Figure 3.

**Figure 4:**
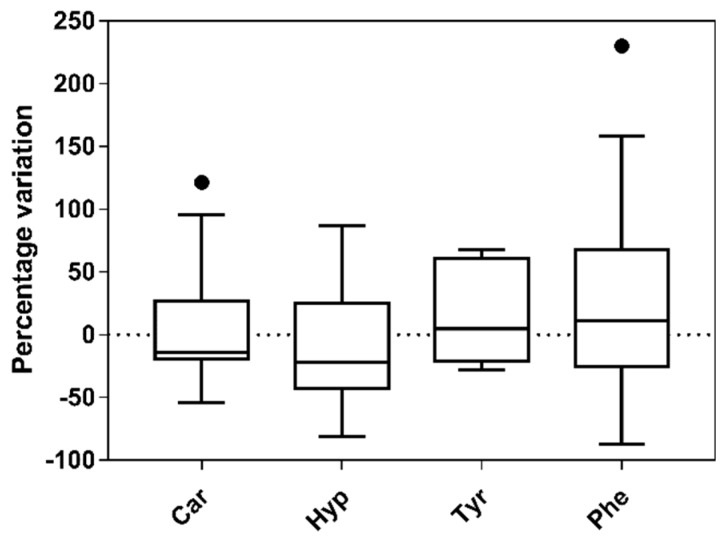
Percentage variation of four biomarkers (Car, carnitine; Hyp, 2-hydroxyhyppurate; Tyr, L-tyrosine; and Phe, phenylalanine), as obtained from NMR spectroscopy applied to the urine samples of patients after EVOO-enriched dark chocolate consumption. (Tukey whiskers are reported, black dots are values greater than the largest value represented by upper whisker).

As is known, the ^1^H NMR spectrum is rather complex [33]. However, several significant signals have been identified, such as carnitine, 2-hydroxyhyppurate, L-tyrosine and phenylalanine. The identification of target signals is based on data available in the literature [23,34]. In particular, we selected the singlet at 3.23 ppm (carnitine), the singlet at 3.99 ppm (2-hydroxyhyppurate), the doublet at 6.88-6.90 ppm (L-tyrosine), and the multiplet at 7.41–7.44 ppm (Phenylalanine). The intensity of NMR signals is proportional to the compound concentration and any intensity variation (positive or negative) is related to a change in the concentration of the metabolite. In our case, we could clearly identify the above signals, in both pre and post treatment, in 18 out of 26 cases for carnitine, 21 out of 26 cases for 2-hydroxyhyppurate, 12 over 26 cases for L-tyrosine and 22 out of 26 cases for phenylalanine. In all other cases, the signals were not above the signal to noise ratio and the relative integrals could not be determined. In Figure 4, the overall percentage variation associated to the four biomarkers is reported. 

Among the four identified metabolites, L-tyrosine was the one determined with high precision (standard deviation on the percentage variation of the NMR signal was about 38%). The worst determination was that of phenylalanine (standard deviation was about 80%), meaning there was a very high variability among patients. By comparing the ^1^H NMR results for all patients, the average value of L-tyrosine increased (median of about 5%), when comparing the pre-treatment with the post-treatment situation; an opposite trend was observed for carnitine and 2-hydroxyhyppurate. In particular, the median for carnitine was −14.5% and the median for 2-hydroxyhyppurate was −22.0%.

## 4. Discussion

Several studies have reported the beneficial effect of high polyphenol cocoa and/or dark chocolate intake on endothelial function as being an improvement in FMD [19,35,36].

The first description of the direct correlation between EPCs and FMD was reported in patients with cardiovascular risk [4]. Subsequent studies have confirmed this relationship, identifying EPCs as either CFU-EC, CD34+KDR+ [37], CD133+KDR+ [38], or CD34+CD133+KDR+ cells [9] in different diseases. 

Heiss et al., first, observed that a 1-month dietary intervention with flavanol-containing cocoa leads to an improvement of endothelial dysfunction associated with an enhanced number and function of circulating angiogenic cells (CD34+/KDR+ -CACs) in patients with CAD [24].

In the present study, based on a sample of subjects with almost 3 cardiovascular risk factors, the main finding was that the consumption of EVOO-enriched dark chocolate (40 g/day, during 4 weeks) significantly improved the circulating EPCs number. We observed that 4-weeks consumption of EVOO-enriched dark chocolate, but not apple-enriched dark chocolate, induces an enhanced number of circulating CD34+CD133+KDR+ cells (EPCs). Despite the elevated concentration of total polyphenols detected in a Panaia red apple-enriched dark chocolate bar, the effect of EVOO-enriched dark chocolate on CD34+CD133+KDR+ cells may be due to the powerful antioxidant capacity. A long list of antioxidant compounds detected in EVOO such as secoiridoids acids, tocopherols etc. (as reported in Supplementary Materials Table S2) could help to improve the health properties of this product.

A number of observational and clinical studies indicated that cocoa or cocoa-containing products may improve blood lipid profile [39], blood pressure [40], or inflammatory status [18,41]. However, not all authors found all of these benefits [17,36]. In our study no significant modification were observed in all biochemical parameters, in agreement with other studies [14,19]. Apple-enriched dark chocolate consumption induced a significant increase in serum LDL cholesterol concentration, however, this result is due to a random effect, as observed after Bonferroni’s correction (*p* = 0.29). The discrepancies in the modulation of lipid profile reflect the results observed in literature, attributable to the amount of flavonoids, the formulation of cocoa products, and the study duration. In our opinion, the differences in the clinical outcome may be due to the different composition in antioxidant compounds. Moreover, both chocolate-type bars do not modulate blood pressure as observed in other papers [42,43], affecting neither glycaemia nor body weight. 

Since the identification of endogenous biomarkers of cocoa could lead to new hypotheses to unravel the relationship between cocoa intake and cardiovascular diseases [29], we focused our attention on the variation of four biomarkers, such as carnitine, 2-hydroxyhyppurate, L-tyrosine and phenylalanine, to evaluate if this analysis could help with early identification of cardiovascular risk markers, highlighting metabolic changes that classical clinical parameters do not immediately detect.

Phenylalanine represents a promising biomarker for early identification of cardiovascular risk, due to the fact that it is a precursor of neurotransmitters strongly associated with CVD in subjects under 60 years old [44]. This amino acid therefore represents a promising biomarker for early identification of cardiovascular risk. In this study, however Phenylalanine determination had a very high variability among patients and for this reason, the mean value of phenylalanine when comparing pre-treatment and post-treatment was not significant. Carnitine is involved in the regulation of long-chain fatty acid metabolism in cardiac muscle. Moreover, it has been proposed that carnitine and its derivatives protect perfused hearts from being subjected to ischaemia and from oxidative stress [45]. In accord with Llorach et al. [29], in our study EVOO-dark chocolate consumption was associated with decreased levels of carnitine metabolism. This reduction may be due to the decreased levels of leucine as observed by Martin et al. [31], after 2 weeks of treatment with dark chocolate. Finally, we observed a decrease in hippurate metabolite. Hippurate, a normal component of urine with a strong association with diet and the intestinal microbiota [46], has been associated with a diverse range of diseases state and metabolic alterations. In particular, the relationship between hippurate excretion and modification in the intestinal microbiota is a new area of investigation, particularly since there is evidence correlating the different microbiomes between obese and lean individuals [47].

## 5. Conclusions

This study shows that 4 weeks consumption of extra virgin olive oil-enriched dark chocolate would improve endothelial function, expressed as an increase in the median levels of circulating endothelial progenitor cells number with potential positive long-term consequences on cardiovascular health. This improvement is due to extra virgin olive oil, since apple-enriched dark chocolate consumption was not effective and in our opinion this effect is probably due to specific extra virgin olive oil polyphenols. 

Moreover, we think that the enhancement in endothelial progenitor cells cannot occur regardless of life style and diet [11], since our study population belongs to an area (Tuscany) with high adherence to the Mediterranean diet.

Regarding changes to metabolites that classical clinical parameters do not immediately detect, recent studies strongly suggest identifying new pathways explaining the benefits of cocoa on cardiovascular [48], and our study supports this hypothesis.

Further investigation is clearly warranted to determine the longer term effects of habitual solid cocoa ingestion and optimal dosing in healthy subjects carrying cardiovascular risk factors. 

## Figures and Tables

**Table 1:** Nutrient composition of the chocolate used in the study (100 g).

Nutrient	Dark Chocolate (70% Cocoa) with 10% Extra Virgin Olive Oil (EVOO)	Dark Chocolate (70% Cocoa) with 2.5% Panaia Red Apple
Energy (kcal)	566	512
Protein (g)	9.2	8.9
Carbohydrate (g)	26.6	39.3
Sugar (g)	24	36.8
Total fat (g)	47	35.3
Saturated fat (g)	24.5	22.1

**Table 2:** Total phenolic content (GAeq, gallic acid equivalents), theobromine, catechin, epicatechin and IC_20_ values of dark chocolate with 10% EVOO or dark chocolate with 2.5% apple studied. Values are the means ± standard deviation (SD) (*n* = 3) on a fresh weight basis. * *p* < 0.05.

Parameters	Dark Chocolate with 10% EVOO	Dark Chocolate with 2.5% Apple	*p* Value
Total Polyphenols (GAeq/100 g fresh weight)	80.9 ± 2.54	130.1 ± 4.80 *	0.000
Theobromine (mg g^−1^ fresh weight)	1.52 ± 0.018 *	1.19 ± 0.004	0.000
Catechin (mg g^−1^ fresh weight)	0.04 ± 0.004 *	0.03 ± 0.005	0.0199
Epicatechin (mg g^−1^ fresh weight)	0.11 ± 0.003 *	0.08 ± 0.003	0.0008
IC_20_ (mg ml^−1^)	2.0 ± 0.10	7.68 ± 0.05 *	0.000

**Table 3:** Baseline characteristics of study population.

Characteristic	Value
N (male/female)	26 (14/12)
Age (years, mean ± SD)	51 ± 9
Body mass index (kg/m^2^)	29 ± 6
**Cardiovascular Risk Factors**	
CAD family history (*n*)	19
Overweight (*n*)	20
Hypertension (*n*)	14
Dyslipidemia (*n*)	15
Active smokers (*n*)	9
**Medication**	
Statins (*n*)	1
ACE-inhibitors (*n*)	3
Beta-blockers (*n*)	2
Calcium channel blockers (*n*)	2
Antidiabetics (*n*)	0
Sartanic (*n*)	3
Diuretic (*n*)	2
Other (*n*)	2

ACE, angiotensin-converting enzyme; CAD, coronary artery disease; Data are expressed as number of patients (*n*) and as mean and SD.

**Table 4:** Biochemical variables at baseline (pre-treatment) and after 4 weeks (post-treatment).

Biochemical Parameter	EVOO-Dark Chocolate Bar		
	Pre-Treatment	Post-Treatment	*p*-Value
BMI (Kg/m^2^)	29 ± 6	29 ± 6	0.80 §
Glucose (mg/dL)	90 ± 9	91 ± 13	0.78 §
Total cholesterol (mg/dL)	216 ± 32	213 ± 33	0.49 ^‡^
HDL cholesterol (mg/dL)	50 ± 13	51 ± 16	0.28 ^‡^
LDL cholesterol(mg/dL)	139 ± 31	133 ± 31	0.09 ^‡^
Triglycerides (mg/dL)	134 ± 61	142 ± 94	0.58 §
Systolic BP (mmHg)	128 ± 13	128 ± 17	0.90 ^‡^
Diastolic BP (mmHg)	84 ± 10	80 ± 9	0.07 ^‡^

BMI: Body mass index; EVOO: extra virgin oilive oil; HDL: High-density lipoprotein, BP: blood pressure; LDL: Low-density lipoprotein. ^‡^ *t*-test. § Wilcoxon test.

**Table 5:** Biochemical variables at baseline (pre-treatment) and after 4 weeks (post-treatment).

Biochemical Parameter	Panaia red Apple-Dark Chocolate Bar		
	Pre-Treatment	Post-Treatment	*p*-value
BMI (Kg/m^2^)	29 ± 6	28 ± 6	0.08 §
Glucose (mg/dL)	91 ± 12	93 ± 8	0.44 §
Total cholesterol (mg/dL)	213 ± 32	220 ± 34	0.16 ^‡^
HDL cholesterol (mg/dL)	50 ± 15	50 ± 14	0.43 ^‡^
LDL cholesterol(mg/dL)	132 ± 32	142 ± 33 *	0.03 ^‡^
Triglycerides (mg/dL)	152 ± 80	135 ± 48	0.37 §
Systolic BP (mmHg)	128 ± 21	123 ± 15	0.18 ^‡^
Diastolic BP (mmHg)	81 ± 10	79 ± 8	0.34 ^‡^

BMI: Body mass index; HDL: High-density lipoprotein, BP: blood pressure, LDL: Low-density lipoprotein. ^‡^ *t*-test. § Wilcoxon test. * *p* < 0.05.

## Supplementary Materials

The following are available online at https://www.mdpi.com/2076-3921/8/4/88/s1, Figure S1: ^1^H NMR spectrum of methanol/water extract of chocolate, Figure S2: Calibration curve of theobromine, Table S1: Polyphenols identification, Table S2: Identification of principal BIOPHENOL compounds in extra virgin olive oil, Table S3: Calibration curves’ parameters for the quantification of theobromine, epicatechin and catechin.
