# Supplementary material for: Effects of Extra Virgin Olive Oil and Apples Enriched-Dark Chocolate on Endothelial Progenitor Cells in Patients with Cardiovascular Risk Factors: A Randomized Cross-Over Trial
Source: Antioxidants (Basel). 2019 Apr 4;8(4):88. doi: 10.3390/antiox8040088 (PMC6523981; doi:10.3390/antiox8040088)
Supplement: Supplementary file 1 [file antioxidants-08-00088-s001.pdf]

Supporting information

1 Theobromine, catechin and epicatechin quantification in chocolate extract by means of ^{1}H NMR

In **Figure S1**, the ^{1}H NMR spectrum of chocolate extract sample is reported. Characteristic signals of theobromine, Catechin and Epicatechin are identified as reported in the figure. Concentration of theobromine, catechin and epicatechin was carried out following the calibration curve method proposed in [Francini *et al.* Food Chemistry, 221:1206–1213].

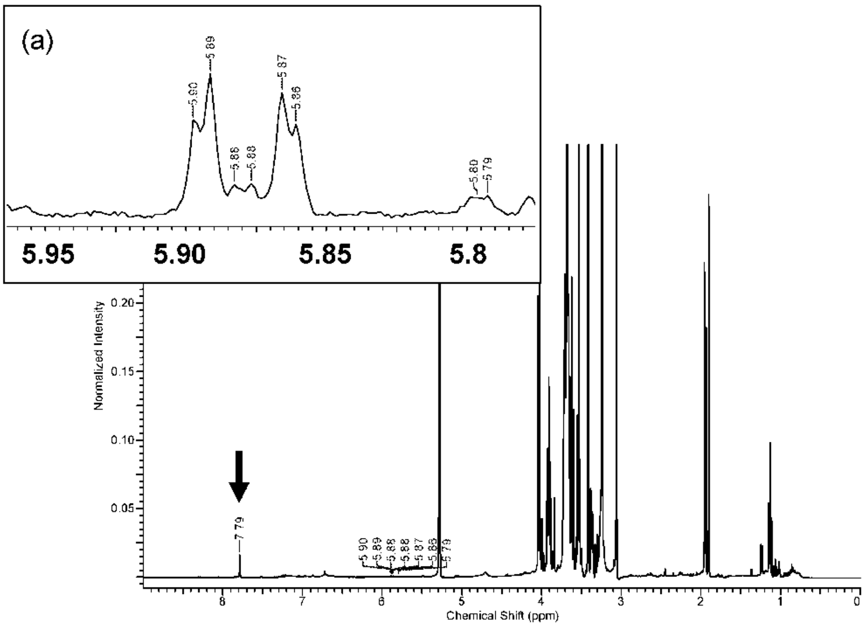

Figure S1. ^{1}H NMR spectrum of methanol/water extract of chocolate. The singlet signal at 7.79 ppm is related to theobromine while signals in the region enlarged in the inset (a) are assigned to catechin, doublets centered at 5.80 and 5.88 ppm, epicatechin doublets, centered at 5.87 and 5.90 ppm.

For each compound, standard solutions of known concentration have been prepared in the same extraction mixture and ^{1}H NMR spectra have been acquired with the same experimental conditions applied to the samples.

2 Polyphenols identification

Identification of four principal polyphenols present in the apples dry samples (chlorogenic acid, phloridzin, (-)-epicatechin and catechin) have been done. Data have been obtained comparing the 1H NMR spectrum of the specific standard with spectra of samples (700 µL) spiked with 100 µl of 1 mg/mL standard solutions.

Table S1. Polyphenols identification.

Polyphenols	mg 100 g^{-1} DW
Chlorogenic acid	13.69±1.548
Phloridzin	8.82±0.348
(-)-epicatechin	3.22±0.635
Catechin	0.94±0.219

3 Characterization of the extracts

Cocoa bars used for this study containing: A) dried apples of cultivar “Panaia” a traditional variety from Tuscany; B) top-quality extra virgin olive oil. Each ingredient was obtained from local organic producers and selected for their high antioxidants levels.

The samples were manufactured in the Vestri chocolate laboratory located in Arezzo (http://www.vestri.it) using the best mixture of cocoa beans produced directly by Vestri in their organic farm in Santo Domingo. All the production conditions (mixing, refining, conching, tempering, molding, and cooling) were set to maintain the highest organoleptic features of the original cocoa beans and ingredients. Bars were packed in 40 g size and stored at 4°C. The composition of the bars was 70% cocoa mass, cane sugar (10%), vanillin (1%), cocoa butter (16.5 % or 9% in apple- or EVOO-enriched bar, respectively), dried Panaia apple (2.5%) or Protected Geographical Indications Tuscan extra virgin olive oil (10%).

Table S2. Identification of principal BIOPHENOL compounds in extra virgin olive oil. Data are expressed as Tyrosol (mg/kg) data are means ± SD.

BIOPHENOL compounds in extra virgin olive oil expressed as Tyrosol	mg/kg
± Hydroxytyrosol	8.7±4
Tyrosol	4.9±2
Vanillic acid + Caffeic acid	1.1±0
Vanillin	2.0±0
p-Coumaric acid	1.0±0
Hydroxytyrosol Acetate (3,4-DHPEA-AC)	4.0±1
Ferulic acid	0.5±0
o-Coumaric acid	0.3±0
Decarboxymethyl oleuropein aglycone, oxidised dialdehyde form	14.9±9
Decarboxymethyl oleuropein aglycone, dialdehyde form (3,4-DHPEA-EDA)	103.7±25
Oleuropein	35.4±7
Oleuropein-aglycone di-aldehyde form (3,4-DHPEA-EA)	5.5±2
Decarboxymethyl ligstroside aglycone, oxidised dialdehyde form	16.4±11
Decarboxymethyl ligstroside aglycone, dialdehyde form (p-HPEA-EDA)	40.6±14
(+)-Pinoresinol ; (+)-1-Acetoxypinoresinol	15.6±1
Cinnamic acid	5.1±1
Ligstroside aglycone, dialdehyde form (p-HPEA-EA)	46.8±7
Oleuropein aglycone, oxidised aldehyde and hydroxylic form	22.4±0
Luteolin	12.2±0
Oleuropein aglycone, aldehyde and hydroxylic form	27.5±10
Ligstroside aglycone, oxidised aldehyde and hydroxylic form	9.0±3
Apigenin	7.4±0
Methyl-luteolin	8.9±2
Ligstroside aglycone, aldehyde and hydroxylic form	1.9±0
TOTAL BIOPHENOLS	396±77
Tocopherols	162±29

Calibration curves have been obtained plotting the sample concentration (mg/mL) versus the integral area, A, of the chosen NMR signals for each component. [Francini *et al.* Food Chemistry, 221:1206–1213].

The calibration curve of theobromine, obtained from its characteristic signal at 7.79 ppm, is reported in **Figure S2**, as an example.

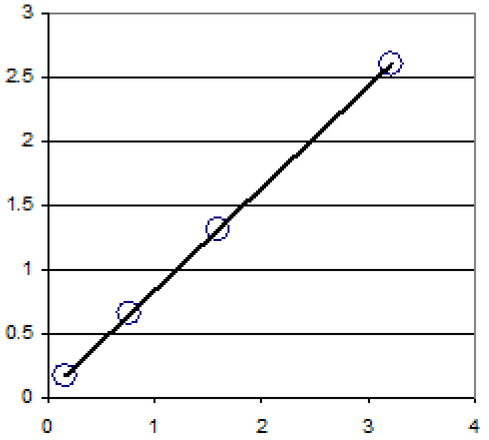

Figure S2. Calibration curve of theobromine. The integral areas, A, calculated as discussed in the text, of the signal at 7.79 ppm in the ^{1}H NMR spectrum of theobromine standard solution, is reported as a function of theobromine concentration (mg/g).

In the linear dynamic range the relation between the integral of the proton signal A and the concentration of the analyte follows the simple equation:

$$A = B[C] + C_0$$

The slope B and C_0 background constant for each compound are reported in the **Table S1** with the values of R^2 for the calibration curves.

Table S3. Calibration curves' parameters for the quantification of theobromine, epicatechin and catechin. Pearson coefficient R^2 is also reported.

Standard	***^{1}H NMR signal (ppm)***	***Coefficient B***	***Coefficient C_0***	***R^2***
Teobromine	*7.79*	*0.7959*	*0.035*	*0.9999*
Epicatechin	*5.86*	*18.9530*	*-0.335*	*0.9987*
Catechin	*5.77*	*7.7080*	*0.005*	*0.9998*
